# Characterization of OT4-1 Alloy by Multi-Dome Forming Test

**DOI:** 10.3390/ma10080899

**Published:** 2017-08-03

**Authors:** Ivan Zakhariev, Sergey Aksenov, Anton Kotov, Aleksey Kolesnikov

**Affiliations:** 1 National Research University Higher School of Economics, Moscow 101000, Russia; aksenov.s.a@gmail.com (S.A.); adkotov@hse.ru (A.K.); 2 Institute of Aircraft Machine Engineering and Transport, National Research Irkutsk State Technical University, Irkutsk 664074, Russia; Avk@istu.edu

**Keywords:** material characterization, mathematical simulation, superplastic materials, titanium alloy, blow forming, multi-dome forming testing, tensile testing, super plastic forming (SPF)

## Abstract

In this study, the rheological characteristics of a titanium alloy have been obtained by multi-dome bulging test. Free bulging process is an experimental technique that can be used to characterize material in conditions of biaxial tension during superplastic, as well as conventional, hot forming. The constitutive constants are calculated on a base of the information about the bulge geometry, applied pressure, and forming time. A multi-dome forming test allows one to reduce the number of the experiments required for the characterization, since every multi-dome test produces several domes of different size. In this study, a specific die for multi-dome test was used. The die has six holes with different radiuses of 20, 25, 30, 35, 40, and 45 mm. During a test, the specimen is clamped between blank holder and die holder, heated to a specific temperature, and formed by applying constant gas pressure. The experiments were conducted at different temperatures for OT4-1 titanium alloy. The constitutive constants were obtained by processing the experimental data using two different techniques and compared with tensile test results. In order to estimate the influence of friction on the experimental results and to verify obtained material characteristics, finite element (FE) simulation was performed. Finally, the results of FE simulation were compared with the experimental data. The results of the simulation show the advantage of material characterization based on multi dome tests and its interpretation by inverse analysis. The deviations produced by the effect of friction are more significant when the direct approach is applied instead of inverse analysis with a semi analytical model of the bulging process.

## 1. Introduction

The characterization of material rheological behavior plays a significant role while the development of forming technologies. It becomes even more important for superplastic forming. A superplasticity effect can be achieved at a narrow range of strain rates which should be provided during the forming process. For some superplastic materials, the elongation at tensile testing could exceed almost 2000% [1]. Low level of flow stress and high strain rate sensitivity of the material are the main features of superplastic deformation. In order to describe material behavior during superplastic deformation, the Backofen constitutive equation [2], using power relation between flow stress $\sigma_{\epsilon }$ and strain rate ${\dot{\varepsilon }}_{\epsilon }$, is commonly used [3,4]

(1) $$\sigma_{\epsilon }=K{\dot{\varepsilon }}_{\epsilon }^{m},$$

where K and m are the material constants. The strain rate sensitivity index m is an important parameter responsible for the stability of plastic flow. A larger m provides better superplasticity. It is considered that the value of m for superplastic materials exceeds 0.5 [4]. Materials with values of m in the range of 0.3–0.5 are considered quasi-superplastic [5].

The most common way to characterize superplastic materials is tensile testing. The results of such tests are simple to interpret. The coefficients of Equation (1) are calculated by the approximation of the measured flow stress values for different strain rates. However, in some investigations [3,6,7,8,9] it was shown that free bulging testing is a more suitable experimental technique for characterization of a material formed in biaxial tension conditions. The comparison of material characteristics obtained by free bulging and tensile tests for aluminum based alloys is presented in [6,7,8,9]. Similar investigations for titanium based alloys are presented in [8]. There are several technics to obtain the material constants based on the results of free bulging tests [10,11,12,13,14,15]. A method for direct calculation of the constants K and m was proposed in [10]. In [11], it was extended to take the strain hardening into consideration. An inverse method based on the finite element (FE) simulation was applied in [12,13,14]. In [15], a special semi-analytical model of the bulging process was developed and used in inverse analysis for solving the direct task instead of finite element method (FEM).

A conventional free bulging test is performed at constant pressure during predetermined time. After testing, the specimen is measured in order to obtain the values of final dome height and the sheet thickness at the dome apex. The amount of information produced by single test can be increased by using specific equipment for real-time measurement of the dome height evolution and application of special forming regimes with stepped pressure changes [12,13]. Another way to increase the informability of a test is to form several domes at once [8,16].

Multi-dome forming technique was first presented by El-Morsy [8] and implemented to obtain superplastic characteristics of titanium and aluminum alloys. The tests were carried using a die with four holes of 20, 25, 30, and 35 mm diameter. The constitutive constants obtained by these tests were compared with the results of uniaxial tensile testing. These results were applied for computer simulation of rectangular box forming [17] to confirm the efficiency of a multi-dome test. Later multi-dome forming tests were used to examine the strain rate sensitivity index of a 7075 Al alloy [16]. In this study, the forming mold has five holes of different diameters: 1, 2, 5, 10 and 15 mm. The experiments were conducted at different temperatures and forming pressures in order to find the optimum forming conditions of 7075 Al alloy. In all these papers, the results of the tests were interpreted using very simple direct methods.

Titanium-aluminum-magnesium OT4-1 alloy is classified as a near alpha alloy. The significant post-uniform deformation of this alloy at ambient and near ambient temperatures was shown in [18]. High temperature superplastic bulge forming of OT4-1 was studied in [19] in a wide range of temperatures. The microstructure of OT4-1 is investigated in [19]. According to this paper the post-formed microstructures show no significant grain size changes.

In this work, the inverse approach proposed in [15,20] was used to interpret the results of the multi-dome forming test. The results were compared with the ones obtained by the direct method in order to study how the interpretation technique could affect the predicted values of the constitutive constants. The effect of friction was studied by finite element simulations of a multi-dome forming process. The Backofen constitutive constants were obtained for titanium alloy OT4-1 both by tensile testing and multi-dome bulging testing.

## 2. Material and Experiments

### 2.1. Material

Titanium alloy OT4-1(Ti-Al-Mn) is the analogue of Japanese ST-A90 Titanium. Its chemical composition is presented in Table 1. This material is widely used in aerospace industry and considered to be superplastic in the temperature range of 800–900 °C [19]. The material flow behavior was investigated by tensile testing at the temperatures of 790, 840, and 890 °C. A series of multi dome forming tests was performed for the temperature of 840 °C.

### 2.2. Tensile Testing

**Figure 1:**
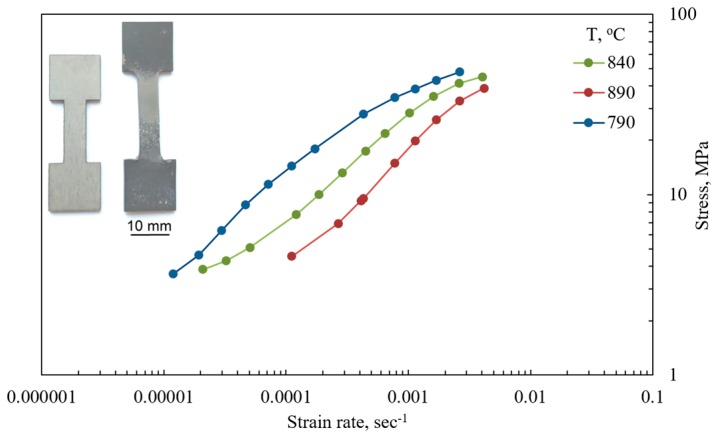
The results of the tests at different temperatures.

A tensile test with a stepped strain rate change was performed in order to estimate the temperature and strain rate conditions of the material superplasticity. The samples have a gauge section size $F_{0}=6\times 1.55$ mm^2^ (width = 6 mm and thickness = 1.55 mm) and length $l_{0}=17$ mm ($l_{0}=5.65\sqrt{F_{0}}$) and were cut parallel to the rolling direction. The specimens were cut from as received OT4-1 sheet and heated for 20 minutes, then deformed in argon atmosphere. The temperature was maintained within an accuracy of ±3°C. The test was performed in the strain rate range $1\times 10^{-5}–5\times 10^{-3}$ s^−1^. The specimen geometry before and after the test performed at the target temperature of 840 °C is presented on Figure 1. In order to observe the effect of temperature on the material behavior, additional tests were performed at 790 and 890 °C. The superplastic behavior was characterized by means of uniaxial tensile tests on a Walter Bay LFM-100 test machine (Walter + Bai AG, Löhningen, Switzerland) with a program service Dion-Pro for the control of the traverse motion in real time. The results of the tests performed at different temperatures are presented on Figure 1. It can be seen that the superplastic strain rate range corresponding to the steep part of a stress strain rate curve plotted in logarithmic scale shifts to the right with increasing temperature.

The stress strain rate curve was approximated using the Backofen equation in order to calculate the values of constitutive constants characterizing OT4-1 alloy behavior at 840 °C in a strain rate range of $1.5\times 10^{-5}–2\times 10^{-3}$ s^−1^.

### 2.3. Multi-Dome Forming Testing

The multi-dome forming tests were performed on the same material machined to rectangular sheet blanks with the sides equal to 320 and 370 mm. The initial thickness of every specimen was 1 mm. The tests were carried out at different constant pressures ($P_{i}$) and forming times ($t_{i,j}$). For each pressure value, three tests were performed with different forming times. The values of pressure and times are presented in Table 2.

**Figure 2:**
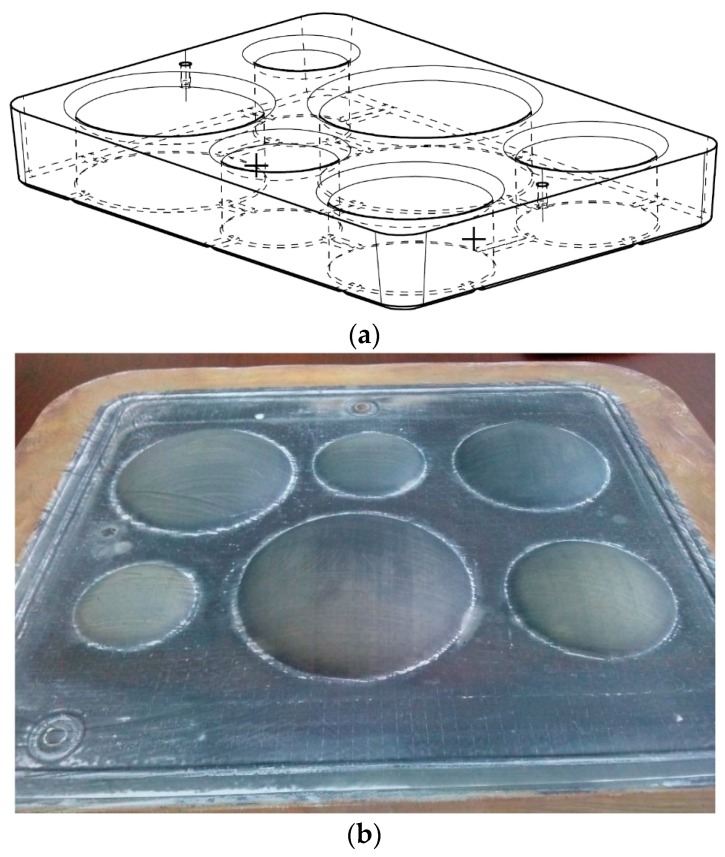
Multi-dome mold (**a**) and the specimen after the test (**b**).

During testing, the formed sheet was clamped between the blank holder with six forming holes and the die holder securing the specimen. The experiments were carried out on the FSP 60T forming machine produced by An Aries Alliance Company (ACB) (Nantes, France). Figure 2a shows the scheme of the mold with forming holes of different diameters: 20, 25, 30, 35, 40, and 45 mm. The photograph of the specimen formed at $P_{1}=0.3\;\mathrm{MPa}$, during $t_{1}=1200\;s$ is presented on Figure 2b.

## 3. Mathematical Model and Characterization Technique

**Figure 3:**
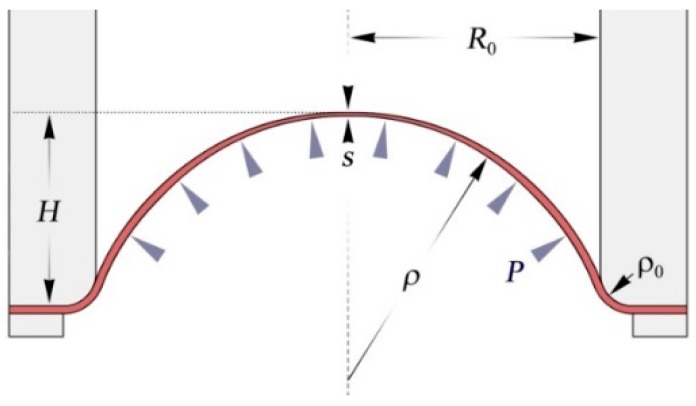
Scheme of free bulging test.

The scheme of the free bulging test is presented on Figure 3. A sheet specimen of initial thickness $s_{0}$ is deformed by pressure P in to a cylindrical die with an aperture radius $R_{0}$ and entry radius $\rho_{0}$, forming a dome. The free part of the dome is assumed to be a spherical surface with radius ρ at an instant moment of time (t). H is the height of the dome and s is the current thickness of the specimen at the dome apex.

The mathematical model describing the forming process is based on following assumptions:

- The material is isotropic
- Elastic strains are negligible
- At any given time, the metal sheet is shaped as a part of the sphere
- The sheet is rigidly clamped

Considering the stress equilibrium of a small element in the dome apex, one can express the value of equivalent stress ($\sigma_{\epsilon }$) as

(2) $$\sigma_{\epsilon }=\frac{P\rho }{2s}.$$

The curvature radius ρ can be expressed as a function of height using the simple geometrical formula

(3) $$\rho \left(H\right)=\frac{H^{2}+{\left(R_{0}+\rho_{0}\right)}^{2}}{2H}-\rho_{0}.$$

Equivalent strain ($\varepsilon_{\epsilon }$) at the dome apex can be expressed as

(4) $$\varepsilon_{\epsilon }=ln\left(\frac{s_{0}}{s}\right).$$

By applying of Equation (2) to the results of multi-dome forming test, one can calculate the value of stress corresponding to each bulge at the final moment of forming. The strain rate corresponding to each stress can be estimated as the value of $\varepsilon_{\epsilon }$ calculated by (4) and divided by forming time ($t_{f}$)

(5) $${\dot{\varepsilon }}_{\epsilon }=\frac{\varepsilon_{\epsilon }}{t_{f}}.$$

The obtained pairs of stress strain rates then can be approximated by Backofen equation. This simple technique was used for material characterization in [3,8]. The drawback is that the Equation (5) gives a very approximate estimation of the strain rate. The strain rate varies significantly during the test and the results based on Equation (5) could become a source of errors.

An alternative technique is based on the mathematical model proposed in [15], allowing one to predict the evolution of a dome height. Using this model, the constitutive constants are calculated by inverse analysis, minimizing the error function constructed as

(6) $$F_{err}=\overset{N}{\underset{i-1}{\sum }}\underset{t}{\min}\left(\sqrt{{\left(\frac{H_{i}\left(t\right)-H_{i}}{H_{i}}\right)}^{2}+{\left(\frac{t-t_{i}}{t_{i}}\right)}^{2}}\right),$$

where N is a number of domes obtained by all experiments, $t_{i}$ and $H_{i}$ are the forming time and the measured height value of the *i*-th dome; $H_{i}\left(t\right)$ is the predicted evolution of the height of the *i*-th dome.

To construct the prediction of a dome height, the stress rate should be expressed as the derivation of the effective strain.

(7) $${\dot{\varepsilon }}_{\epsilon }=\frac{d}{dt}\left(ln\left(\frac{s_{0}}{s}\right)\right)=-\frac{1}{s}\frac{ds}{dt}=-\frac{1}{s}\frac{ds}{dH}\frac{dH}{dt},$$

where the value of apex thickness is considered to be a function of dome height [20]

(8) $$s=s_{0}\left(1-B\frac{H}{\rho +\rho_{0}}\right).$$

Combining the Equations (2), (7), and (8) with Equation (1), one can construct the differential equation for dome height evolution

(9) $$\frac{dH_{i}}{dt}=\frac{\left(\rho \left(H_{i}\right)+\rho_{0}-B_{i}H_{i}\right)\left(H_{i}\left(\rho \left(H_{i}\right)+\rho_{0}\right)\right)}{B_{i}{\left(R_{0}+\rho_{0}\right)}^{2}}{\left(P\rho \left(H_{i}\right)\right)}^{1/m}{\left[2Ks_{0}\left(1-B_{i}\frac{H_{i}}{\rho \left(H_{i}\right)+\rho_{0}}\right)\right]}^{-1/m}$$

The parameter $B_{i}$ can be calculated based on the experimental results as

(10) $$B_{i}=\left(\frac{s_{i}}{s_{0}}-1\right)\left(\frac{H_{i}{}^{2}+{\left(R_{0}+\rho_{0}\right)}^{2}}{2H_{i}{}^{2}}\right),$$

where $s_{i}$ is the experimental thickness at the dome apex.

## 4. Results and Discussion

### 4.1. Material Characterization

**Figure 4:**
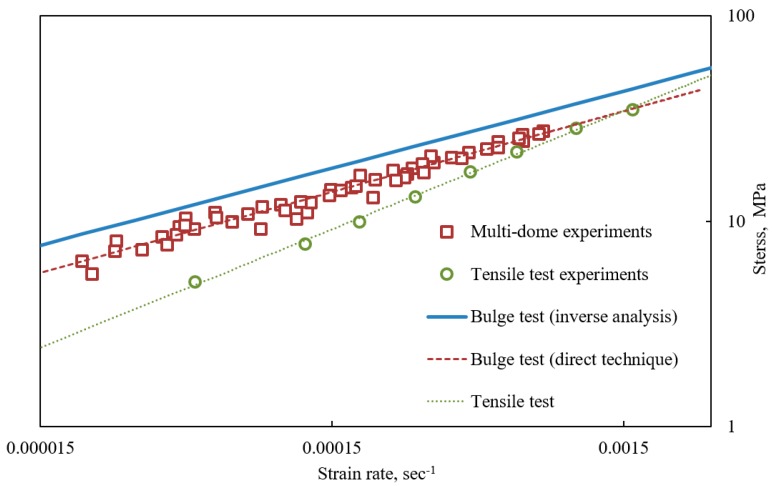
Results of multi-dome forming tests, tensile tests, and constitutive equations obtained by their processing, plotted in logarithmic scale.

The processing of multi-dome forming results was performed using two interpretation techniques. According to the simple direct technique described in Section 3, five pairs of effective stress $\sigma_{\epsilon }$ and effective strain rate ${\dot{\varepsilon }}_{\epsilon }$ values for each of nine tests were calculated using Equations (2) and (5). The obtained 45 ($\sigma_{\epsilon }$, ${\dot{\varepsilon }}_{\epsilon }$) points were fitted by the power equation shown on Figure 4 and the constitutive constants were found as: K=444, m=0.394.

Comparing these results with the tensile test data which were approximated by Backofen equation with the constants K=1471 and m=0.577, significant deviations can be observed. The differences between the constitutive constants obtained using different experimental techniques were noticed in many studies [6,7,8,9]. The results of free bulging tests are considered to be more convenient for simulation of SPF while they are obtained in conditions of biaxial stress tension which are close to the ones realized in production.

Inverse analysis of the multi dome forming results were performed in two steps. At first, for every dome apex point ($H_{i}$, $s_{i}$) on every specimen, the coefficient $B_{i}$ was calculated by Equation (10). The objective function $F_{err}$ was constructed according to Equation (6) using numerical solution of Equation (9) for the evaluation of $H_{i}\left(t\right)$. The Backofen constants were found to be those corresponding to the minimum of $F_{err}$ at the values of K=494 and m=0.375.

The comparison of constitutive data obtained by different techniques is presented on Figure 4. The results of inverse analysis are plotted by the blue solid line. The ($\sigma_{\epsilon }$, ${\dot{\varepsilon }}_{\epsilon }$) points obtained by the direct method are illustrated by square red markers. The power fitting of these points is plotted by the red dashed line. The green markers and the green dotted line illustrate the results obtained by the tensile test. It can be seen that, in the given strain rate, the multi-dome forming tests produce higher stresses and lower strain rate sensitivity than the tensile ones.

### 4.2. Finite Element Verification

**Figure 5:**
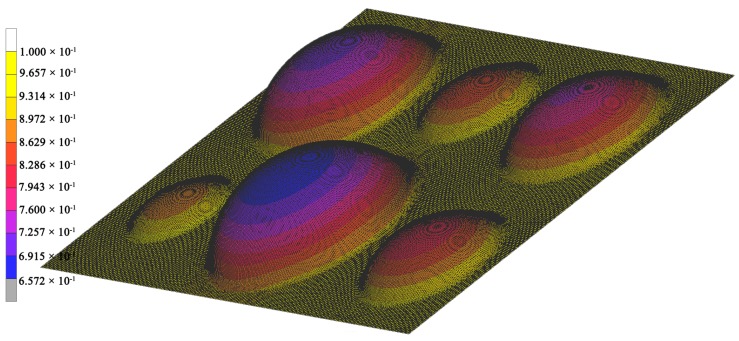
Thickness distribution after multi-dome forming FE simulation.

The constitutive constants obtained by different methods were verified by finite element (FE) simulation of the multi-dome forming process. Three-dimensional simulation was carried out by MSC. Marc software (Newport Beach, CA, USA). A finite element mesh was generated using four-node regular plane elements. The deformable sheet of an initial thickness of 1 mm was divided into 195,520 elements. The simulations were made for all the pressures used in the experiments. The pressure and temperature were considered to be constant in every simulation. The die was simulated as a rigid undeformable body. Three different pairs of constitutive constants obtained by the techniques described above were used for simulation of material properties. The results of FE simulation of multi-dome forming at 0.3 MPa during 3600 s are presented on Figure 5. The color field illustrates thickness distribution within the specimen.

**Figure 6:**
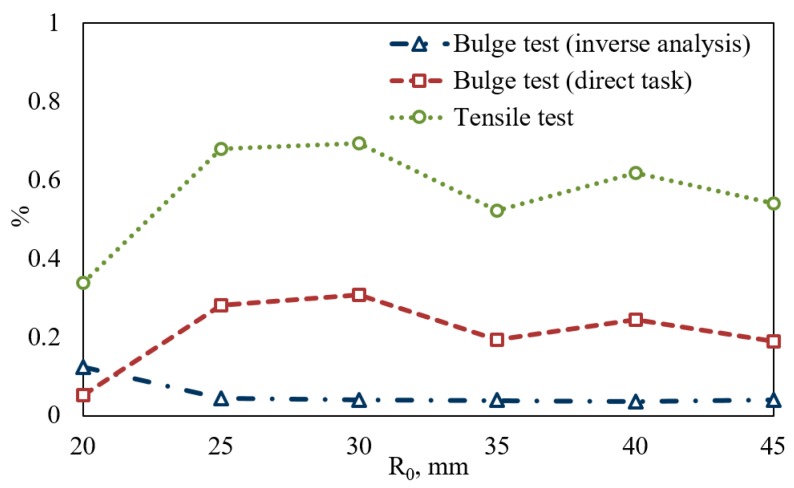
Deviation between experimental data and FE simulations.

The comparison of the reults of FE simulation with the experimental data is presented in Table 3 and Figure 6. Table 3 contains the values of mean deviation between the measured height and FE predictions made for different pressures. The curves plotted on Figure 6 illustrate the dependence of relative error averaged by all forming pressures and times on the radius of the bulge. It can be seen that inverse analysis allows one to obtain much better results than other techniques. The larger deviations from measured values are observed in simulations made in which constitutive equations are obtained by tensile testing.

### 4.3. Effect of Friction

In order to estimate the influence of friction on the accuracy of the results obtained by interpretation of multi-dome forming tests an additional series of calculations with different values of friction factor was performed. FE simulations were carried out with the following friction coefficient values: 0.1, 0.3, 0.5, 0.7, and 0.9. The calculations were proceed with material characteristics obtained from inverse analysis (K=494, m=0.375). The values of pressure and forming time were set at $P_{1}=0.3\;\mathrm{MPa}$, $t_{1}=4000\;s$;$\;P_{2}=0.5\;\mathrm{MPa}$, $t_{2}=1500\;s$; $P_{3}=0.7\;\mathrm{MPa}$, $t_{3}=700\;s$.

**Figure 7:**
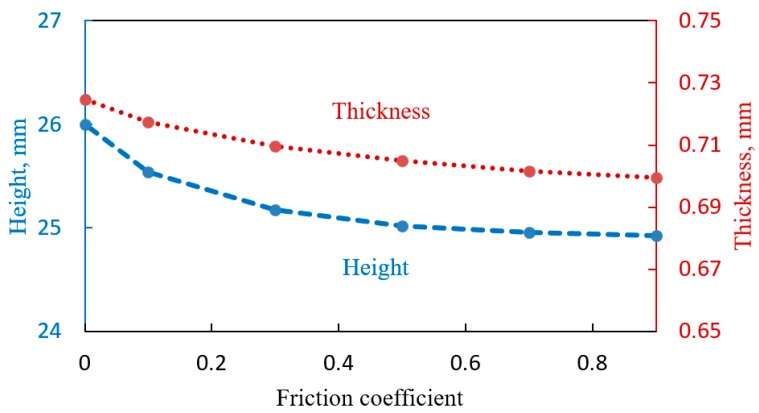
Variations in height and thickness with friction at equal moment of time and pressure regime.

Analyzing the results of finite element simulation, one can estimate how the effect of friction affects the results of multi-dome bulging test. Considering a single dome with maximum aperture radius ($R_{0}=45\;\mathrm{mm}$) at a fixed moment of time and pressure (t=4000 s, P=0.3 MPa) it is possible to notice the monotonic decrease of height and thickness at the dome apex with increasing friction as it is plotted on Figure 7.

**Figure 8:**
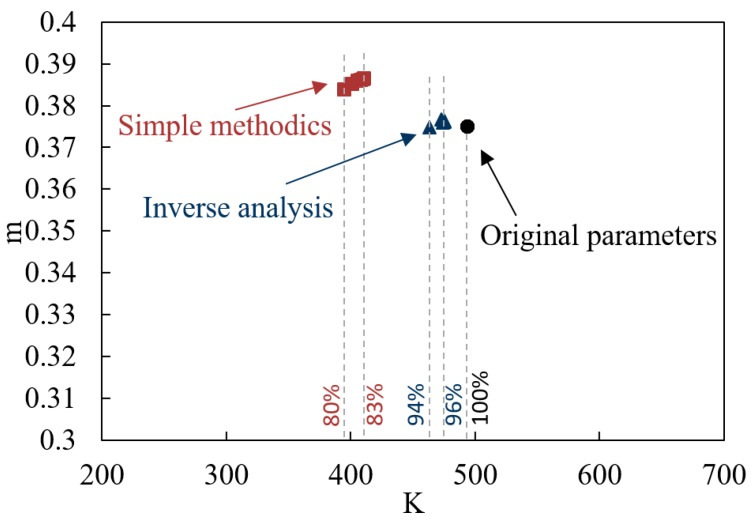
K,m parameters, obtained by FE simulation processing.

The values of height and thickness of the specimen at each bulge calculated by FEM were used as the initial data both for direct and inverse techniques. The constitutive constants obtained by this method were compared with the ones which were used in the simulations. This information allows one to estimate how large the difference could be between the results of interpretation of multi-dome forming test and the real material constants, and how this difference is affected by friction and interpretation technique. The results of comparison are illustrated on Figure 8.

The red quadric markers on Figure 8 correspond to the parameters K, m obtained by application of Equations (2) and (5) to the results of FE simulations and their approximation by Backofen power law according to the direct method. The blue triangle markers correspond to the results of inverse analysis and the black marker corresponds to the initial parameters. It can be seen that the values of strain rate sensitivity (m), obtained by inverse analysis are very close to the referenced one. The values of m calculated by direct method are generally higher than the referenced one at 0.01–0.015, which can be treated as a neglectable error in most cases. The deviations of K are more significant and reach 20% for the direct method and 6% for the inverse one. The effect of friction on the deviations between the calculated constitutive constants and the reference constants is more significant when the direct approach is applied.

## 5. Conclusions

In this study, the multi-dome forming process is studied numerically and experimentally. Backofen constitutive constants OT4-1 titanium alloy were evaluated by multi-dome forming tests using both direct and inverse techniques. The results were compared with the data obtained by tensile testing and verified by finite element simulation. The deviations produced by the effect of friction on the experimental results were estimated for both direct and inverse methods.

The results of experimental data processing point to a difference of material characteristics obtained from tensile tests and free bulging experiments. For the OT4-1 alloy at 840 ºC, the stress values calculated from multidome tests are higher than those obtained from tensile tests for the same strain rates. It was shown that the inverse analysis based on a semi analytical model of free bulging process allows one to perform more accurate interpretation of the results of a multi-dome forming test than the direct approach. At the same time, using the results of tensile testing with stepped changing of strain rate may lead the appearance of large errors in the simulation of SPF processes.

## Figures and Tables

**Table 1:** Chemical composition of OT4-1 alloy.

Fe %	C %	Si %	Mn %	N %	O %	Al %	Zr %	Ti %
0.3	0.1	0.12	2	0.05	0.15	2.5	0.3	Balance

**Table 2:** Time values for different pressures.

Pressure, MPa	$t_{1}$, s	$t_{2}$, s	$t_{3}$, s
0.3	1200	2400	3600
0.5	400	800	1200
0.7	180	260	540

**Table 3:** Deviations between FE-simulations and multi-dome tests.

P, MPa	Equation	Deviations					
		$R_{0}=20$	$R_{0}=25$	$R_{0}=30$	$R_{0}=35$	$R_{0}=40$	$R_{0}=45$
0.3	Inverse analysis	0.1501	0.0521	0.0367	0.0199	0.0299	0.0333
0.3	Direct technique	0.0371	0.3106	0.3117	0.1984	0.2349	0.1829
0.3	Tensile test	0.4556	1.006	0.9658	0.7084	0.8454	0.7349
0.5	Inverse analysis	0.1532	0.0177	0.0291	0.0345	0.0237	0.0332
0.5	Direct technique	0.0204	0.2470	0.2978	0.1584	0.2258	0.1665
0.5	Tensile test	0.2779	0.5881	0.6641	0.4550	0.5617	0.4861
0.7	Inverse analysis	0.0653	0.0604	0.0566	0.0623	0.0564	0.0515
0.7	Direct technique	0.0971	0.2875	0.3125	0.2245	0.2726	0.2196
0.7	Tensile test	0.2799	0.4412	0.4532	0.4012	0.4443	0.3976
